# The Effect of User Psychology on the Content of Social Media Posts: Originality and Transitions Matter

**DOI:** 10.3389/fpsyg.2020.00526

**Published:** 2020-04-21

**Authors:** Lucia Lushi Chen, Walid Magdy, Maria K. Wolters

**Affiliations:** School of Informatics, University of Edinburgh, Edinburgh, United Kingdom

**Keywords:** affect, social media, emotion, Facebook, personality traits, depression, mental health, non-original content

## Abstract

Multiple studies suggest that frequencies of affective words in social media text are associated with the user's personality and mental health. In this study, we re-examine these associations by looking at the transition patterns of affect. We analyzed the content originality and affect polarity of 4,086 posts from 70 adult Facebook users contributed over 2 months. We studied posting behavior, including silent periods when the user does not post any content. Our results show that more extroverted participants tend to post positive content continuously and that more agreeable participants tend to avoid posting negative content. We also observe that participants with stronger depression symptoms posted more non-original content. We recommend that transitions of affect pattern derived from social media text and content originality should be considered in further studies on mental health, personality, and social media.

## 1. Introduction

Many people express rich moods and emotions in their social media posts. Psychologists use the word “affect” to describe these experiences of feelings and emotions. Affect plays an important role in cognition (Gross et al., 1998) and well-being (Silvera et al., 2008). Therefore, affective expressions in social media text have emerged as a key variable for making inferences about users' personality traits (Golbeck et al., 2011; Bachrach et al., 2012; Farnadi et al., 2013) or mental health (De Choudhury et al., 2013; Coppersmith et al., 2014; De Choudhury and De, 2014; Bazarova et al., 2015).

Existing studies formulate the associations between affect and well-being based on the frequencies of affective words used in social media text (Yarkoni, 2010; Golbeck et al., 2011; Schwartz et al., 2013; Park et al., 2015; Chen et al., 2020). However, patterns of affect are an important class of symptoms of affective disorders (Frijda, 1993; Rottenberg, 2005; Bylsma et al., 2011; Carlo et al., 2012; Thompson et al., 2012; Houben et al., 2015; Sheppes et al., 2015). Personality may also predispose individuals to specific moods (Rusting and Larsen, 1995; Rusting, 1998). With this in mind, we examined how patterns of affect expressed in social media text are related to users' mental health and personality.

While non-original content has been extensively studied in opinion mining (Balahur et al., 2009; Agarwal et al., 2011), it has been comparatively neglected in the study of psychological interpretations of social media data. However, social media users often use lyrics or quotes to communicate their emotions. Such content comes from other media, such as literature, videos, films, or music, which can evoke strong emotional experiences (Scherer and Zentner, 2001; Juslin and Laukka, 2004; Scherer, 2004). Since the affect of the non-original content may be different from the social media users' affect when they are post this content, we differentiated between original and non-original content in our analysis.

This pilot study was designed to examine the following research questions:

1. **Changes in Affect:** To what extent do changes in the affect of social media posts correlate with users' personality traits and mental well-being?
2. **Originality:** To what extent does the use of non-original material in their posts correlate with users' personality traits and mental well-being?

Following best practice in sentiment analysis and opinion mining, we distinguish between positive, negative, neutral, and mixed (both positive and negative) affect (Moilanen and Pulman, 2007; Agarwal et al., 2011; Rosenthal et al., 2015).

We used a well-known dataset, myPersonality (Bachrach et al., 2012; Youyou et al., 2015), which enriches Facebook posts with many validated psychological measures. In MyPersonality, positive mental well-being is measured using the Satisfaction with Life Scale (Diener et al., 1985, 1999), while the presence of depressive symptoms is assessed using the Centre for Epidemiologic Studies Depression scale (CES-D) (Radloff, 1977). Personality traits are established following the OCEAN model (McCrae and John, 1992), which consists of the five traits Openness to Experience, Conscientiousness, Extroversion, Agreeableness, and Neuroticism.

We included all 70 adult users who provided sufficient, regular Facebook data for 2 months before completion of the CES-D questionnaire and corrected for multiple comparisons in our statistical analysis. We find that the transitions from one affective state to another expressed in social media posts give us a highly nuanced view of personality traits. While the amount of non-original posts in ones' social media status updates is closely linked to depression symptoms, this link is mediated by neuroticism.

## 2. Background

Affect refers to both mood and emotion. Moods are slow-moving states that can be influenced by people, objects or situations, whereas emotions are quick reactions to stimuli (Watson, 2000; Rottenberg and Gross, 2003) and are highly situation- or object-specific (Bylsma et al., 2008). Mood influences the probability of having emotions of the same valence—negative mood facilitates negative emotions, and positive mood makes positive emotions more likely (Fredrickson, 1998; Rottenberg, 2005). Affect is an important predictor of mental well-being, including a person's overall satisfaction with life (Headey et al., 1993; Singh and Jha, 2008; Chen et al., 2017), and the level of symptoms of depression (Coppersmith et al., 2015; Resnik et al., 2015; Tsugawa et al., 2015).

Personality also predisposes people to certain affective states (Rothbart et al., 2000). While neuroticism is associated with negative affect (Pishva et al., 2011), positive affect is strongly linked to extroversion (Fujita et al., 1991; Watson and Clark, 1997). Extroverts experience more positive affect because they engage in more social situations (Diener and Emmons, 1984; Ryan and Deci, 2001). Individuals who score high on agreeableness have a greater ability to regulate negative affect (Meier et al., 2006; Haas et al., 2007). This relationship between affect and personality is also reflected in social media studies (Pennebaker and King, 1999; Golbeck et al., 2011; Schwartz et al., 2013; Lin et al., 2017). For example, people who use negative affective words in their social media posts tend to have lower conscientiousness, lower agreeableness (Golbeck et al., 2011), and higher neuroticism (Pennebaker and King, 1999).

In psychology, quantitative representations of affect are typically multidimensional (Russell, 1980). In this study, we focus on valence, which is represented in many classic affect models. Traditional measures, such as the Positive and Negative Affect Schedule (PANAS) (Watson et al., 1988), report the strength of positive and negative valence. Mixed valence can occur when people experience “dialectic” emotion, which is a mix of positive and negative emotions (Schimmack et al., 2002; Russell, 2003).

The personality trait measurements in myPersonality are based on Costa and McCrae's well-validated OCEAN model (McCrae and John, 1992). The model consists of five dimensions: extroversion, agreeableness, conscientiousness, neuroticism, and openness to experience. Neuroticism refers to the degree of emotional stability. Openness reflects the degree of creativity and curiosity. Conscientious individuals tend to be careful and diligent. Extroversion refers to a tendency to be energetic and friendly. Agreeableness reflects the tendency to be compassionate and to cooperate with others (Digman, 1990). The five-factor structure has proved to be robust in both self and peer ratings (McCrae and John, 1992), in both children and adult (Mervielde et al., 1995), and across different cultures (McCrae and Allik, 2002) and to be stable over time (McCrae and John, 1992).

## 3. Data and Methodology

The myPersonality data set (Bachrach et al., 2012; Youyou et al., 2015) contains more than 180,000 Facebook users, enriched with a variety of additional validated scales (Bachrach et al., 2012). The collection of myPersonality data complied with the terms of service of Facebook, informed consent for research use was obtained from all users, and researchers had to seek permission to use the dataset. Permission for the use of this database was obtained before it closed for new studies in 2018. The study was granted Ethical Approval by the Ethics Committee of the School of Informatics, University of Edinburgh.

### 3.1. Choice of Scales

From the extensive data collected within myPersonality, we chose two scales for quantifying mental well-being, the *Center for Epidemiologic Studies Depression Scale (CES-D)* and the *Satisfaction with Life Scale (SWL)*. The CES-D scale measures a key aspect of mental health, the presence of depression symptoms (Radloff, 1977). The scale has high internal consistency, test-retest reliability (Radloff, 1977; Roberts, 1980; Orme et al., 1986), and validity (Orme et al., 1986). Following previous social media studies (Park et al., 2012; De Choudhury et al., 2013), we adopt a score of 22 or higher as a cut-off value for likely depressive disorder (maximum score: 60). The five-item SWL scale has been tested across different cultures and age groups (Pavot and Diener, 2009) and has been found to have high internal consistency and temporal reliability (Diener et al., 1985). Personality traits were measured using a 100-item scale using items from the open-source International Personality Item Pool (Goldberg et al., 2006) that were validated against the NEO-PI-R (Schwartz et al., 2013) instrument.

### 3.2. Selection of Participants

The data set was originally designed for a study of the effect of mental well-being and values on social media disclosure. We therefore selected only those participants who had completed the CES-D scale, the SWL scale, and the Schwartz Value survey (Schwartz, 1992) in addition to the full personality questionnaire. A total of 301 participants in myPersonality provided full data for all four scales.

To ensure we had enough posts to assess the frequency of affect transitions, we only included users in our sample that regularly updated their public Facebook feed (*regular users*). We defined regular users as individuals who posted on average twice a week or more. We estimated posting frequency using the average post-count per day during the sampling frame. If an individual had a post-count per day of 0.3, this individual made around 110 posts in 365 days, which was roughly equivalent to an average of two posts per week. Of the original 301 participants, 122 (40.5%) were regular users.

Since the CES-D asks about symptoms in the past week, we excluded a further 31 users who had not posted any content in the week before completing the CES-D scale. We then focused on a 60-days span (2 months) before CES-D completion to ensure that we had sufficient data to track the development of users' moods. We removed 14 users who contributed <20 posts during that time. Finally, we removed four users who were under 18 years old and three users with more than 20% of the posts written in a language other than English, because English was the common language of the annotation team. The final sample consisted of 4,086 posts from 70 users.

### 3.3. Corpus Annotation

#### 3.3.1. Social Media Affect

For the purpose of this study, we refer to the affect shown in social media posts as *social media affect*. In this study, following (Mohammad, 2016), we operationalize valence as the post-author's attitude toward a primary target of opinion. We refer to the “dialectic” affective state as *mixed valence*. If there is no clear trend toward positive or negative affect, the associated valence is *neutral*.

After extensive piloting, we created an annotation guideline (available as part of the supplementary material) that was largely based on Mohammad (2016)'s work on defining the valence of a social media post. Each post is assigned one of four affect polarities: + (positive), − (negative), ± (mixed), or 0 (neutral). We used manual annotation since this is commonly used in computational linguistics to create a baseline gold standard data set for further analysis (Teufel, 1999).

Of the 4,086 posts, 2,698 (66%) were annotated by a team of six trained annotators and 1,185 (29%) by the first author; 5% of all posts were annotated by all seven annotators to establish inter-rater reliability, which was measured using Cohen's κ (Gamer et al., 2019). Average inter-rater reliability between the first author and the annotators is 0.88, and it is 0.78 among the six annotators.

After annotation, most of the posts were of positive valence (*N* = 1,588, 39%), followed by negative valence (*N* = 1,164, 28%), neutral valence (*N* = 982, 24%), and mixed valence (*N* = 312, 8%). A total of 40 posts were excluded from analysis because they did not contain English text.

#### 3.3.2. Originality

We define posts that consist of quotes from sources, such as song lyrics, books, or movies as non-original content; all other content was defined as original. Since non-original content might not directly reflect the user's moods or emotions, annotators were instructed to annotate such posts according to the likely emotions of the author. For example, if a post consists of an uplifting motivational quote, annotators considered the underlying valence to be positive.

In order to establish the originality of a post, we retrieved the first page of results obtained by searching for the post-text using the Google API. For each web page on the first page of results, we computed the cosine similarity between the post-content and the page content. Posts with a cosine similarity >0.96 were labeled as non-original, and posts with a cosine similarity between 0.92 and 0.96, where the website links or website names included the words “lyrics” or “quote” were labeled as potentially non-original. Posts with a cosine similarity lower than 0.92 were labeled as original. The cutoff points were determined based on a sample of 300 posts manually annotated for originality by the first author. On these posts, the classifier yields 100% recall, 81% precision, and an F1-score of 0.89. In our data set, 287 (7%) of all posts were identified as non-original.

### 3.4. Modeling Affect Transitions

We examine two types of transitions:

- **Post-level vs. Day-level:** *Post-level* transitions focus on changes in affect between subsequent social media posts, whereas *day-level* transitions focus on changes in overall dominant affect between subsequent days.
- **Silence vs. Non-silence:** Not all users post every day. In our *default* models, these silent days are ignored, whereas in our *with-silence* models, days without posts are explicitly modeled as *Silence*.

The post-level social media affect is likely to be influenced by *underlying emotions*, which change more quickly, whereas the day-level social media affect is likely to be influenced by *underlying mood* during the day. Day-level affect was calculated as follows. If the majority of the posts *p*_*ij*_ on day *d*_*j*_ have the same affect *a*, then the affect of day *d*_*j*_ is set to *a*. If there is an equal number of positive (+) and negative (−) posts or if the number of mixed affect (±) posts is equal to the number of posts with other types of affect, affect is set to ± (mixed). For transitions between original and non-original posts, we only consider the post-level representation. Table 1 shows an example of the affect and originality representations.

**Table 1:** Affect and originality representation for a sample week.

	**Monday**	**Tuesday**	**Wednesday**	**Thursday**	**Friday**	**Saturday**	**Sunday**
**Affect**							
Post-level	+ –	+ – +	+ +	S	±	0	+ –
Day-level	–	+	+	S	±	0	±
**Originality**							
Post-level	O N O	O O N	N N	S	O	O	N N

*↔; −, negative valence; +, positive valence; ±, mixed valence; S, silence day; O, original content; N, non-original content*.

### 3.5. Statistical Analysis

Demographic differences between users above and below the CES-D cut-off score for probable depression were assessed using Wilcoxon-Mann-Whitney tests (R-package “Stats”).

We used Pearson correlation coefficients to assess the significance of correlations between social media data on the one hand and personality traits and mental well-being on the other hand. Due to the small sample size and the number of correlations computed, all correlation coefficients were estimated using a permutation approach (Higgins, 2003), as implemented in the R Package jmuOutlier (Garren, 2017). Correlations that reach *p* < 0.01 or better are reported as significant; correlations that reach *p* < 0.05 are reported as trends in the data. For all correlations reported in the paper, we give the estimated correlation coefficient, the bootstrap 95% confidence interval, and the corresponding coefficient of determination *r*^2^.

## 4. Results

### 4.1. Demographics and Baseline Statistics

Table 2 shows the basic statistics of our sample. Our data predominantly comes from single female Caucasian young adults. The average CES-D score is above the cut-off for possible depressive disorder.

**Table 2:** Demographics of the sample.

**Variable**	***N* (%)**	**Variable**	**Mean (SD)**
**Gender**		**Age**	
- Female	49 (70%)	- Female	23.52 (6.56)
- Male	21 (30%)	- Male	22.84 (7.13)
**Ethnicity**		**Personality**	
- Caucasian	54 (75%)	- Openness to Experience	4.19 (0.46)
- Black	3 (4%)	- Conscientiousness	3.20 (0.75)
- Asian	5 (7%)	- Extraversion	3.11 (3.83)
- Other	8 (14%)	- Agreeableness	3.55 (0.68)
		- Neuroticism	2.98 (0.89)
**Living status**		**Mental well-being**	
- Living with partner	8 (10%)	- SWL	4.18 (1.44)
- Single	54 (77%)	- CES-D	23.79 (11.86)
- Married	5 (7%)		
- Unknown	3 (4%)		

*Caucasian includes White people of American, British, and other origins; Black includes African-Americans and Black people from Europe. SWL, score for Satisfaction with Life Scale; CES-D, Center for Epidemiologic Studies Depression Scale*.

Thirty-nine (56%) participants had a CES-D score of 22 or higher (mean: 33, SD: 6.5), which means that it is possible that they have depressive disorder, and 31 (44%) had a score of 21 or lower (mean: 12, SD: 6).

**Figure 1:**
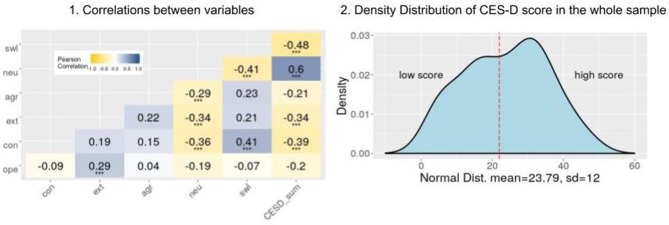
Basic statistics for personality trait scores, SWL and CES-D scores. Plot 1 is a heat map of correlations between personality traits, SWL, and CES-D scores (^***^p < 0.001). Plot 2 illustrates the distribution of the CES-score in the entire sample (*N* = 70). The dotted line indicates the cutoff score of 22.

Participants with possible depressive disorder are less extroverted (*Z* = 375, *p* < 0.005) and have higher levels of neuroticism (*Z* = 990, *p* < 0.001), lower levels of conscientiousness (*Z* = 375, *p* < 0.001), and lower satisfaction with life (*Z* = 323, *p* < 0.001). Detailed results are reported in Figure 1 Plot 1.

All scales are normally distributed (Shapiro-Wilks test), except for openness to experience (*W* = 0.96, *p* < 0.05) and satisfaction with life (*W* = 0.95, *p* < 0.05), which are bimodal. Figure 1 Plot 1 shows the correlations between different personality dimensions. As expected, the five personality dimensions are not orthogonal.

### 4.2. Social Media Affect: Frequencies vs. Transitions

For **overall frequencies of affect category**, the only clear correlation is between extroversion and positive content. Overall, more extroverted participants are more likely to have days where they make predominantly positive posts (*r* = 0.29, *p* < 0.01, 95%CI = (−0.15, 0.32), *r*^2^ = 0.08). In addition, participants who score higher on agreeableness tend to post fewer negative posts and have fewer days with predominantly negative posts [both *r* = −0.26, *p* < 0.05, 95%CI = (−0.48, −0.04), *r*^2^ = 0.07].

When we look at **transitions between affect categories**, however, a more nuanced picture emerges. Table 3 summarizes the correlations between personality, well-being and transition types. Significant correlations are summarized in Table 4. Due to the number of correlations presented, we choose a cut-off of *p* < 0.01, which is stricter than the normal *p* < 0.05.

**Table 3:** Correlations between personality, SWL, and CES-D scores and affect transitions. Number of participants *N* = 70.

					**Post-level representation (post-plus silence)**										
	S↔S	−↔−	+↔+	±↔±±↔±	0↔0	+↔−	±↔+	±↔−	±↔0	0↔+	0↔−	S↔+	S↔−	±↔S	S↔0
N_*Occ*_	1238	346	542	29	230	599	143	134	100	424	414	641	384	137	211
ope	0.09	−0.17	−0.17	−0.16	−0.05	−0.14	−0.07	−0.08	0.11	0.01	0.03	0.17	0.00	0.13	0.03
con	−0.06	0.01	0.09	−0.09	−0.15	0.11	0.00	−0.01	−0.14	−0.07	−0.08	0.16	0.00	0.15	−0.15
ext	0.04	−0.12	0.16	−0.10	−0.19	−0.06	−0.03	−0.12	−0.09	−0.09	−0.17	0.29^**^	−0.04	0.00	−0.18
agr	0.14	−0.37^***^	0.03	0.02	−0.15	−0.22^*^	0.08	0.04	0.04	−0.04	−0.23^*^	0.23^*^	−0.04	0.29^**^	−0.13
neu	−0.07	0.19	0.18	0.18	−0.03	0.23^*^	0.11	0.04	0.02	0.05	−0.05	−0.22^*^	−0.03	−0.23^*^	−0.13
swl	0.04	−0.10	−0.13	−0.10	0.06	−0.03	0.02	−0.05	−0.04	0.02	−0.08	0.02	0.16	−0.02	0.18
CESD	−0.04	0.19	0.08	0.09	0.00	0.04	0.15	0.07	0.03	−0.06	0.11	−0.20·	0.00	−0.11	−0.03
					**Post-level representation (post-only)**, ***N*** **= 70**										
N_*Occ*_		396	694	34	313	728	188	166	142	547	502				
ope		−0.16	−0.05	−0.06	−0.02	−0.05	0.06	−0.01	0.14	0.09	0.13				
con		−0.07	0.18	−0.07	−0.23^*^	0.08	0.14	0.10	−0.11	−0.13	−0.12				
ext		−0.04	0.33^***^	0.04	−0.24^*^	0.05	0.08	−0.10	−0.15	−0.16	−0.20·				
agr		−0.28^**^	0.18	0.00	−0.16	−0.10	0.26^*^	0.28^**^	0.13	0.03	−0.26^*^				
neu		0.14	0.00	0.11	−0.02	0.16	−0.14	−0.09	−0.08	0.01	−0.12				
swl		0.00	−0.12	−0.11	0.11	0.02	0.09	0.09	−0.02	0.08	−0.04				
CESD		0.14	−0.04	0.03	0.04	−0.03	−0.06	−0.11	0.04	−0.11	0.13				
					**Day-level representation**, ***N*** **= 70**										
N_*Occ*_	228	281	271	267	304	287	303	296	298	261	311	242	259	261	261
ope	0.12	−0.17	−0.11	−0.05	−0.02	−0.08	0.00	−0.14	−0.07	−0.01	−0.02	0.12	−0.02	0.19	0.13
con	−0.06	−0.03	0.25^*^	0.05	−0.01	0.03	−0.03	−0.04	−0.16	−0.19	−0.12	0.08	0.10	0.06	−0.07
ext	0.06	−0.11	0.30^***^	−0.03	−0.14	0.04	0.14	−0.13	0.01	−0.28^**^	−0.28^**^	0.24^*^	−0.08	0.02	−0.17
agr	0.11	−0.22	0.15	−0.05	0.08	−0.12	0.16	−0.06	0.11	−0.08	−0.17	0.15	−0.07	0.28^**^	−0.09
neu	−0.08	0.16	0.00	0.19	−0.17	0.21^*^	0.09	0.11	−0.01	0.12	0.08	−0.14	−0.12	−0.26^*^	−0.03
swl	0.02	−0.08	−0.01	−0.08	0.25^*^	−0.03	−0.06	−0.10	0.03	−0.06	−0.04	−0.02	0.12	0.06	0.08
CESD	−0.03	0.11	−0.10	0.08	−0.18	0.02	0.10	0.08	0.08	−0.01	0.21·	−0.18	0.03	−0.16	0.05

Pearson correlation P-value (permutation testing): · < 0.1,

* < 0.05,

** < 0.01,

*** *< 0.001, bidirectional transition types: ↔; −, negative valence; +, positive valence; ±, mixed valence; 0, neutral; S, silence day; N_Occ_, number of occurrences of each transition type; ope, openness; con, conscientiousness; ext, extraversion; agr, agreeableness; neu, neuroticism; swl, satisfaction with life scale; CESD, Center for Epidemiologic Studies Depression Scale. Bold: p < 0.05*.

**Table 4:** Summary of the significant correlations between transition states and the five personality traits (*p* < 0.01).

	**Transitions**	**Post-level** **(with-silence)**	**Post-level** **(without-silence)**	**Day-level**
Extraversion	S ↔ +	↑	–	–
	0 ↔ +	–	–	↓
	0 ↔ –	–	–	↓
	+ ↔ +	–	↑	↑
Agreeablness	– ↔ –	↓	↓	–
	±↔ S	↑	–	↑
	±↔ –	–	↑	–

*↓ Indicates a significant negative correlation at p < 0.01 or better, ↑ indicates a significant positive correlation at p < 0.01 or better. — Indicates that the correlation is not significant at this level. Bidirectional transition types: ↔; −, negative valence; +, positive valence; ±, mixed valence; 0, neutral; S, silence day*.

Several transition types are correlated positively and negatively with Extroversion and Agreeableness. Neuroticism, conscientiousness, and SWL show interesting trends (*p* < 0.05) that do not reach significance (c.f. Table 3).

More extroverted participants are more likely to post predominantly positive content several days in a row [*day-level*, +↔+, *r* = 0.30, *p* < 0.001, 95% CI = (0.06, 0.54), *r*^2^ = 0.09]. They have more transitions to or from a silence day with a positive post [*post-level with-silence*, S↔+, *r* = 0.29, *p* < 0.01, 95% CI = (−0.01, 0.46), *r*^2^ = 0.08]. This pattern fits well with the overall predominance of posts with positive affect. Extroverts are also less likely to alternate between days with neutral and days with non-neutral content [*day-level*, for both 0↔+ and 0↔−, *r* = −0.28, *p* < 0.01, 95% CI = (−0.52, −0.09), *r*^2^ = 0.08].

People who score higher on agreeableness are less likely to follow a post with negative affect with another negative-affect post [−↔−, *post-level with-silence*: *r* = −0.37, *p* < 0.001, 95% CI = (−0.50, −0.06), *r*^2^ = 0.14]. This tendency is much less pronounced on the day level [−↔−, *r* = −0.22, *p* < 0.1, 95% CI = (−0.44, −0.02), *r*^2^ = 0.04]. On top of that, they are more likely to alternate between days with mixed valence and silence [*day-level*, ±↔S, *r* = 0.28, *p* < 0.01, 95% CI = (−0.01, 0.46), *r*^2^ = 0.08, post-level with-silence, ±↔S, *r* = 0.29, *p* < 0.01, 95% CI = (0.08, 0.52), *r*^2^ = 0.08].

Participants with higher neuroticism tend to alternate between positive and negative content, but this is only evident when we take silence into account [+↔−, *post-level with-silence*: *r* = 0.23, *p* < 0.05, 95% CI = (0.00, 0.47), *r*^2^ = 0.04, *post-level without-silence*: *r* = 0.16, 95% CI = (−0.08, 0.41), *r*^2^ = 0.025, *day-level*: *r* = 0.21, *p* < 0.05, 95% CI = (−0.46, −0.10), *r*^2^=0.04].

There are interesting differences in transition patterns that incorporate information about silence days and those that do not. When disregarding silence days, we observe that people with higher conscientiousness or extroversion are slightly less likely to follow a neutral post with another neutral post [*post-level without-silence*, conscientiousness, 0↔0, *r* = −0.23, *p* < 0.05, 95% CI = (−0.41, −0.04), *r*^2^ = 0.07; extroversion, 0↔0, *r* = −0.24, *p* < 0.05, 95% CI = (−0.41, −0.04), *r*^2^ = 0.07].

When we take into account silence days for computing transitions, we find several more interesting trends. People who are more satisfied with life are more likely to follow a neutral post with another neutral post [0↔0, *day-level*: *r* = 0.25, *p* < 0.05, 95% CI = (−0.01, 0.44), *r*^2^ = 0.06]. In addition, people with higher neuroticism are more likely to alternate between positive and negative posts [0↔−, *day-level*: *r* = 0.21, *p* < 0.05, 95% CI = (−0.01, 0.40), *r*^2^ = 0.04] but less likely to make a positive post after one or more silence days [S↔+, *post-level with-silence*: *r* = −0.22, *p* < 0.05, 95% CI = (−0.48, 0.00), *r*^2^ = 0.04]. We found that silence-to-silence transitions are not correlated with personality or mental health.

### 4.3. Post-originality

High CES-D scores are significantly correlated with posting non-original content [*r* = 0.29, *p* < 0.01, 95% CI = (0.10, 0.46), *r*^2^ = 0.08]. There is a similar tendency for participants with higher neuroticism scores [*r* = 0.25, *p* < 0.05, 95% CI = (0.06, 0.43), *r*^2^ = 0.07]. Examining transitions between post-originality shows that these effects stem from slightly different posting patterns. Users with higher CES-D scores tend to follow non-original content with non-original content [N↔N, *post-level with-silence, r* = 0.26, *p* < 0.05, 95% CI = (0.07, 0.43), *r*^2^ = 0.07] or to alternate between original and non-original content [N↔O *post-level with-silence, r* = 0.27, *p* < 0.05, 95% CI = (0.08, 0.44), *r*^2^ = 0.07]. Users with higher neuroticism scores tend to post-sequences of non-original content [N↔N, post-level with-silence, *r* = 0.25, *p* < 0.05, 95% CI = (0.06, 0.43), *r*^2^ = 0.05] and are less likely to post-original content before or after a period of silence [O↔S, *post-level with-silence, r* = 0.28, *p* < 0.05, 95% CI = (0.09, 0.45), *r*^2^ = 0.08].

Since neuroticism is closely linked to depression symptoms, we also computed a partial correlation between content originality and CES-D while controlling for neuroticism. The resulting correlation was no longer significant (*r* = 0.14, *p* = 0.22, *r*^2^ = 0.02). Therefore, the association between content originality and depression symptoms might be moderated by neuroticism.

## 5. Discussion

### 5.1. Main Findings

Many studies have found associations between the frequency of affective words used in social media text and personality. However, existing studies often saw affect as static and only focused on the strength of bipolar valence (positive/negative). Instead, our work focuses on affect patterns. We encode posting behavior, transitions between affect states, and content originality. From a practical point of view, our technique can supplement experience sampling techniques (Myin-Germeys et al., 2018) to help clinicians and patients develop a more comprehensive view of a person's affect patterns, arrive at a better-substantiated diagnosis, and make improved treatment decisions. However, this depends on whether the patient is willing to share information from their social media feed with their therapist. Overall, the correlations seen between affect transitions and personality traits are in line with the consensus in the early literature (Gross et al., 1998). Extroverts tend to produce sequences of positive posts. This behavior fits well with the positive emotional core in extroverts stipulated in (Watson and Clark, 1997). Participants with higher agreeableness are less likely to post-sequences of negative posts. This could be due to their ability to regulate negative affect (Meier et al., 2006; Haas et al., 2007).

Although the psychology literature suggests a strong association between negative mood states and neuroticism (Rusting and Larsen, 1995), we did not find this in our data. Our results are in line with previous studies of verbal cues to personality traits in social media (Yarkoni, 2010; Golbeck et al., 2011; Schwartz et al., 2013; Park et al., 2015). Golbeck et al. (2011) found that social media users who were more likely to talk about anxiety were on the higher end of the neuroticism scale. We speculate that self-presentation bias may influence how social media users regulate their expression of negative emotions in their public posts. The only relevant association we found was that social media users on the high end of neuroticism are more likely to switch between posting positive and negative affective content. This finding aligns well with the fact that high neuroticism is associated with high emotional instability (Costa and McCrae, 1992).

The link between posting non-original content and elevated depression symptoms appears to be moderated by neuroticism. This suggests that high levels of neuroticism predispose users both to depressive symptoms and to an indirect disclosure of emotions through quotes and lyrics.

In our sample, the prevalence of depressive symptoms is higher than would be expected in the general population. In the original CES-D paper, Radloff (1977) proposed three levels of depression severity: low (0–15), mild-to-moderate (16–22), and high (23–60). They found that only 21% of the general population scored above the low symptom level. In contrast, in our sample, nearly half of the participants exhibit a high level of symptoms (>22). Within the context of social media studies of depression, however, our data set is not exceptional. For many studies in the area, high symptom individuals account for nearly half of the data set (De Choudhury et al., 2013; Tsugawa et al., 2015; Nadeem, 2016; Reece et al., 2017; Orabi et al., 2018).

Our results support the claim that affect expressed in social media data text is associated with social media users' affect patterns in real life. However, the data set used in this study is from the early 2010's and only covers the well-established social media platform Facebook. The associations found in this study are likely to be slightly different from those found in another social networks (e.g., Instagram) or in a new data set collected 10 years later.

### 5.2. Limitations

Due to the restrictions imposed by the need for sufficient Facebook updates to allow analysis, our final sample is relatively small. Given the size of the significant effects we found in the data, power calculations indicate that a well-powered study should include data from around 200 users (Schönbrodt and Perugini, 2013). It also skews heavily toward younger female Caucasians with relatively low satisfaction with life and strong depression symptoms. It is possible that other groups of users (e.g., non-Caucasians, males) are less likely to disclose personal information about mood and emotions on their public Facebook pages (Dosono et al., 2017; McDonald et al., 2019).

## 6. Conclusion and Future Work

In this pilot study, we demonstrated the benefits of detailed representations of social media affect for unpacking the relationship between personality, mental well-being, and the content posted on social media. Importantly, our representations include non-binary affect categories (positive, negative, mixed, neutral), and take into account content originality. As a consequence, we were able to obtain a more detailed picture of the link between patterns of affect and depressive symptoms.

In future work, we plan to enrich our data set with more in-depth analyses of original vs. non-original content, extend coverage by including a larger sample of the myPersonality data set, and construct statistical models that allow us to observe long-term trends in posting patterns. Future studies should also examine the extent to which affect expressed in non-original content is aligned with the users' affect when they post the material.

## Data Availability Statement

The datasets generated for this study will not be made publicly available because the myPersonality database is closed for further research. Requests to access the datasets should be directed to the corresponding author.

## Ethics Statement

The studies involving human participants were reviewed and approved by Self-Certification according to the procedure of the School of Informatics, University of Edinburgh. The patients/participants provided their written informed consent to participate in this study. The secondary analysis of this data set was reviewed and approved by the Ethics Committee of the School of Informatics, University of Edinburgh, Reference Number 72771.

## Author Contributions

LC: study design, statistical analysis, analysis of results, and drafting of paper. WM: principal supervisor of LC. MW: second supervisor of LC. WM and MK contributed to paper writing, advised on study design, statistical analysis, and analysis of results.

## Conflict of Interest

The authors declare that the research was conducted in the absence of any commercial or financial relationships that could be construed as a potential conflict of interest.

## References

[B1] Agarwal A. Xie B. Vovsha I. Rambow O. Passonneau R. (2011). Sentiment analysis of twitter data, in Proceedings of the Workshop on Language in Social Media (LSM 2011) (Portland, OR), 30–38.

[B2] Bachrach Y. Kosinski M. Graepel T. Kohli P. Stillwell D. (2012). Personality and patterns of facebook usage, in Proceedings of the 4th Annual ACM Web Science Conference (New York, NY: ACM), 24–32. 10.1145/2380718.2380722

[B3] Balahur A. Steinberger R. Goot E. v. d. Pouliquen B. Kabadjov M. (2009). Opinion mining on newspaper quotations, in Proceedings of the 2009 IEEE/WIC/ACM International Joint Conference on Web Intelligence and Intelligent Agent Technology, Vol. 3 (Milano: IEEE Computer Society), 523–526. 10.1109/WI-IAT.2009.340

[B4] Bazarova N. N. Choi Y. H. Schwanda Sosik V. Cosley D. Whitlock J. (2015). Social sharing of emotions on facebook: channel differences, satisfaction, and replies, in Proceedings of the 18th ACM Conference on Computer Supported Cooperative Work & *Social Computing* (Vancouver, BC: ACM), 154–164. 10.1145/2675133.2675297

[B5] Bylsma L. M. Morris B. H. Rottenberg J. (2008). A meta-analysis of emotional reactivity in major depressive disorder. Clin. Psychol. Rev. 28, 676–691. 10.1016/j.cpr.2007.10.001 18006196

[B6] Bylsma L. M. Taylor-Clift A. Rottenberg J. (2011). Emotional reactivity to daily events in major and minor depression. J. Abnorm. Psychol. 120:155. 10.1037/a0021662 21319928

[B7] Carlo G. Mestre M. V. McGinley M. M. Samper P. Tur A. Sandman D. (2012). The interplay of emotional instability, empathy, and coping on prosocial and aggressive behaviors. Pers. Individ. Differ. 53, 675–680. 10.1016/j.paid.2012.05.022

[B8] Chen L. Cheng C. H. K. Gong T. (2020). Inspecting vulnerability to depression from social media affect. Front. Psychiatry 11:54. 10.3389/fpsyt.2020.00054 32153438 PMC7047149

[B9] Chen L. Gong T. Kosinski M. Stillwell D. Davidson R. L. (2017). Building a profile of subjective well-being for social media users. PLoS ONE 12:e0187278. 10.1371/journal.pone.0187278 29135991 PMC5685571

[B10] Coppersmith G. Dredze M. Harman C. (2014). Quantifying mental health signals in twitter, in Proceedings of the Workshop on Computational Linguistics and Clinical Psychology: From Linguistic Signal to Clinical Reality (Denver, CO), 51–60. 10.3115/v1/W14-3207

[B11] Coppersmith G. Dredze M. Harman C. Hollingshead K. Mitchell M. (2015). Clpsych 2015 shared task: depression and ptsd on twitter, in Proceedings of the 2nd Workshop on Computational Linguistics and Clinical Psychology: From Linguistic Signal to Clinical Reality (Denver, CO), 31–39. 10.3115/v1/W15-1204

[B12] Costa P. T. McCrae R. R. (1992). Neo Pi-R. Odessa, FL: Psychological Assessment Resources.

[B13] De Choudhury M. De S. (2014). Mental health discourse on reddit: Self-disclosure, social support, and anonymity, in Eighth International AAAI Conference on Weblogs and Social Media (Michigan), 21–30.

[B14] De Choudhury M. Gamon M. Counts S. Horvitz E. (2013). Predicting depression via social media, in Seventh International AAAI Conference on Weblogs and Social Media (Cambridge, MA), 170–185.

[B15] Diener E. Emmons R. A. (1984). The independence of positive and negative affect. J. Pers. Soc. Psychol. 47:1105. 10.1037/0022-3514.47.5.1105 6520704

[B16] Diener E. Suh E. M. Lucas R. E. Smith H. L. (1999). Subjective well-being: three decades of progress. Psychol. Bull. 125:276 10.1037/0033-2909.125.2.276

[B17] Diener E. D. Emmons R. A. Larsen R. J. Griffin S. (1985). The satisfaction with life scale. J. Pers. Assess. 49, 71–75. 10.1207/s15327752jpa4901_13 16367493

[B18] Digman J. M. (1990). Personality structure: emergence of the five-factor model. Annu. Rev. Psychol. 41, 417–440. 10.1146/annurev.ps.41.020190.002221

[B19] Dosono B. Rashidi Y. Akter T. Semaan B. Kapadia A. (2017). Challenges in transitioning from civil to military culture: hyper-selective disclosure through ICTs. Proc. ACM Hum. Comput. Interact. 1, 41:1–41:23. 10.1145/3134676

[B20] Farnadi G. Zoghbi S. Moens M.-F. De Cock M. (2013). Recognising personality traits using facebook status updates, in Seventh International AAAI Conference on Weblogs and Social Media (Cambridge, MA), 154–164.

[B21] Fredrickson B. L. (1998). What good are positive emotions? Rev. Gen. Psychol. 2, 300–319. 10.1037/1089-2680.2.3.300 21850154 PMC3156001

[B22] Frijda N. H. (1993). Moods, emotion episodes, and emotions. Handb. Emot. 12:155.

[B23] Fujita F. Diener E. Sandvik E. (1991). Gender differences in negative affect and well-being: the case for emotional intensity. J. Pers. Soc. Psychol. 61:427. 10.1037/0022-3514.61.3.427 1941513

[B24] Gamer M. Lemon J. Fellows I. Singh P. (2019). irr: Various Coefficients of Inter-Rater Reliability and Agreement. R package version 0.84.81. Available online at: https://cran.r-project.org/web/packages/irr/irr.pdf

[B25] Garren S. T. (2017). Permutation Tests for Nonparametric Statistics Using R. Asian J. Math. 5, 1–8.

[B26] Golbeck J. Robles C. Edmondson M. Turner K. (2011). Predicting personality from twitter, in 2011 IEEE Third International Conference on Privacy, Security, Risk and Trust and 2011 IEEE Third International Conference on Social Computing (IEEE), 149–156. 10.1109/PASSAT/SocialCom.2011.33

[B27] Goldberg L. R. Johnson J. A. Eber H. W. Hogan R. Ashton M. C. Cloninger C. R. (2006). The international personality item pool and the future of public-domain personality measures. J. Res. Pers. 40, 84–96. 10.1016/j.jrp.2005.08.007

[B28] Gross J. J. Sutton S. K. Ketelaar T. (1998). Relations between affect and personality: support for the affect-level and affective-reactivity views. Pers. Soc. Psychol. Bull. 24, 279–288. 10.1177/0146167298243005

[B29] Haas B. W. Omura K. Constable R. T. Canli T. (2007). Is automatic emotion regulation associated with agreeableness? A perspective using a social neuroscience approach. Psychol. Sci. 18, 130–132. 10.1111/j.1467-9280.2007.01861.x 17425532

[B30] Headey B. Kelley J. Wearing A. (1993). Dimensions of mental health: life satisfaction, positive affect, anxiety and depression. Soc. Indicat. Res. 29, 63–82. 10.1007/BF01136197

[B31] Higgins J. (2003). Introduction to Modern Non-Parametric Statistics. The American Statistician. Pacific Grove, CA, 61:184 10.1198/tas.2007.s81

[B32] Houben M. Van Den Noortgate W. Kuppens P. (2015). The relation between short-term emotion dynamics and psychological well-being: a meta-analysis. Psychol. Bull. 141:901. 10.1037/a0038822 25822133

[B33] Juslin P. N. Laukka P. (2004). Expression, perception, and induction of musical emotions: a review and a questionnaire study of everyday listening. J. New Music Res. 33, 217–238. 10.1080/0929821042000317813

[B34] Lin J. Mao W. Zeng D. D. (2017). Personality-based refinement for sentiment classification in microblog. Knowl. Based Syst. 132, 204–214. 10.1016/j.knosys.2017.06.031

[B35] McCrae R. R. Allik J. (Eds.). (2002). The Five-Factor Model of Personality Across Cultures. New York, NY: Springer Science and Business Media.

[B36] McCrae R. R. John O. P. (1992). An introduction to the five–factor model and its applications. J. Pers. 60, 175–215. 10.1111/j.1467-6494.1992.tb00970.x 1635039

[B37] McDonald J. Harris K. L. Ramirez J. (2019). Revealing and concealing difference: a critical approach to disclosure and an intersectional theory of closeting. Commun. Theory 30, 84–104. 10.1093/ct/qtz017

[B38] Meier B. P. Robinson M. D. Wilkowski B. M. (2006). Turning the other cheek: agreeableness and the regulation of aggression-related primes. Psychol. Sci. 17, 136–142. 10.1111/j.1467-9280.2006.01676.x 16466421

[B39] Mervielde I. Buyst V. Fruyt F. D. (1995). The validity of the big-five as a model for teachers' ratings of individual differences among children aged 4–12 years. Pers. Individ. Differ. 18, 525–534. 10.1016/0191-8869(94)00175-R

[B40] Mohammad S. (2016). A practical guide to sentiment annotation: challenges and solutions, in Proceedings of the 7th Workshop on Computational Approaches to Subjectivity, Sentiment and Social Media Analysis (San Diego, CA), 174–179. 10.18653/v1/W16-0429

[B41] Moilanen K. Pulman S. (2007). Sentiment composition, in Proceedings of the Recent Advances in Natural Language Processing International Conference (Borovets), 378–382.

[B42] Myin-Germeys I. Kasanova Z. Vaessen T. Vachon H. Kirtley O. Viechtbauer W.. (2018). Experience sampling methodology in mental health research: new insights and technical developments. World Psychiatry 17, 123–132. 10.1002/wps.20513 29856567 PMC5980621

[B43] Nadeem M. (2016). Identifying depression on twitter. arXiv 1607.07384.

[B44] Orabi A. H. Buddhitha P. Orabi M. H. Inkpen D. (2018). Deep learning for depression detection of twitter users, in Proceedings of the Fifth Workshop on Computational Linguistics and Clinical Psychology: From Keyboard to Clinic (New Orleans, LA), 88–97.

[B45] Orme J. G. Reis J. Herz E. J. (1986). Factorial and discriminant validity of the center for epidemiological studies depression (ces-d) scale. J. Clin. Psychol. 42, 28–33. 10.1002/1097-4679(198601)42:1<28::AID-JCLP2270420104>3.0.CO;2-T 3950011

[B46] Park G. Schwartz H. A. Eichstaedt J. C. Kern M. L. Kosinski M. Stillwell D. J.. (2015). Automatic personality assessment through social media language. J. Pers. Soc. Psychol. 108:934. 10.1037/pspp0000020 25365036

[B47] Park M. Cha C. Cha M. (2012). Depressive moods of users portrayed in twitter, in Proceedings of the ACM SIGKDD Workshop on Healthcare Informatics (HI-KDD), Vol. 2012 (New York, NY: ACM), 1–8.

[B48] Pavot W. Diener E. (2009). Review of the satisfaction with life scale. Psychol. Assess. 5:164 10.1037/1040-3590.5.2.164

[B49] Pennebaker J. W. King L. A. (1999). Linguistic styles: Language use as an individual difference. J. Pers. Soc. Psychol. 77:1296. 10.1037/0022-3514.77.6.1296 10626371

[B50] Pishva N. Ghalehban M. Moradi A. Hoseini L. (2011). Personality and happiness. Proc. Soc. Behav. Sci. 30, 429–432. 10.1016/j.sbspro.2011.10.084

[B51] Radloff L. S. (1977). The CES-D scale: a self-report depression scale for research in the general population. Appl. Psychol. Meas. 1, 385–401. 10.1177/014662167700100306

[B52] Reece A. G. Reagan A. J. Lix K. L. Dodds P. S. Danforth C. M. Langer E. J. (2017). Forecasting the onset and course of mental illness with twitter data. Sci. Rep. 7:13006. 10.1038/s41598-017-12961-9 29021528 PMC5636873

[B53] Resnik P. Armstrong W. Claudino L. Nguyen T. Nguyen V.-A. Boyd-Graber J. (2015). Beyond LDA: exploring supervised topic modeling for depression-related language in twitter, in Proceedings of the 2nd Workshop on Computational Linguistics and Clinical Psychology: From Linguistic Signal to Clinical Reality (Denver, Co), 99–107. 10.3115/v1/W15-1212

[B54] Roberts R. E. (1980). Reliability of the ces-d scale in different ethnic contexts. Psychiatry Res. 2, 125–134. 10.1016/0165-1781(80)90069-4 6932058

[B55] Rosenthal S. Nakov P. Kiritchenko S. Mohammad S. Ritter A. Stoyanov V. (2015). Semeval-2015 task 10: sentiment analysis in twitter, in Proceedings of the 9th International Workshop on Semantic Evaluation (SemEval 2015) (Denver, CO), 451–463. 10.18653/v1/S15-2078

[B56] Rothbart M. K. Ahadi S. A. Evans D. E. (2000). Temperament and personality: origins and outcomes. J. Pers. Soc. Psychol. 78:122. 10.1037/0022-3514.78.1.122 10653510

[B57] Rottenberg J. (2005). Mood and emotion in major depression. Curr. Direct. Psychol. Sci. 14, 167–170. 10.1111/j.0963-7214.2005.00354.x

[B58] Rottenberg J. Gross J. J. (2003). When emotion goes wrong: realizing the promise of affective science. Clin. Psychol. Sci. Pract. 10, 227–232. 10.1093/clipsy.bpg012

[B59] Russell J. A. (1980). A circumplex model of affect. J. Pers. Soc. Psychol. 39:1161 10.1037/h0077714

[B60] Russell J. A. (2003). Core affect and the psychological construction of emotion. Psychol. Rev. 110:145. 10.1037/0033-295X.110.1.145 12529060

[B61] Rusting C. L. (1998). Personality, mood, and cognitive processing of emotional information: three conceptual frameworks. Psychol. Bull. 124:165. 10.1037/0033-2909.124.2.165 9747185

[B62] Rusting C. L. Larsen R. J. (1995). Moods as sources of stimulation: relationships between personality and desired mood states. Pers. Individ. Differ. 18, 321–329. 10.1016/0191-8869(94)00157-N

[B63] Ryan R. M. Deci E. L. (2001). On happiness and human potentials: a review of research on hedonic and eudaimonic well-being. Annu. Rev. Psychol. 52, 141–166. 10.1146/annurev.psych.52.1.141 11148302

[B64] Scherer K. R. (2004). Which emotions can be induced by music? What are the underlying mechanisms? And how can we measure them? J. New Music Res. 33, 239–251. 10.1080/0929821042000317822

[B65] Scherer K. R. Zentner M. R. (2001). Emotional effects of music: production rules. Music Emot. Theory Res. 361:392.

[B66] Schimmack U. Oishi S. Diener E. (2002). Cultural influences on the relation between pleasant emotions and unpleasant emotions: Asian dialectic philosophies or individualism-collectivism? Cogn. Emot. 16, 705–719. 10.1080/02699930143000590

[B67] Schönbrodt F. D. Perugini M. (2013). At what sample size do correlations stabilize? J. Res. Pers. 47, 609–612. 10.1016/j.jrp.2013.05.009

[B68] Schwartz H. A. Eichstaedt J. C. Kern M. L. Dziurzynski L. Ramones S. M. Agrawal M.. (2013). Personality, gender, and age in the language of social media: the open-vocabulary approach. PLoS ONE 8:e0073791. 10.1371/journal.pone.0073791 24086296 PMC3783449

[B69] Schwartz S. H. (1992). Universals in the content and structure of values: theoretical advances and empirical tests in 20 countries, in Advances in Experimental Social Psychology, Vol. 25 (Cambridge, MA: Elsevier), 1–65. 10.1016/S0065-2601(08)60281-6

[B70] Sheppes G. Suri G. Gross J. J. (2015). Emotion regulation and psychopathology. Annu. Rev. Clin. Psychol. 11, 379–405. 10.1146/annurev-clinpsy-032814-112739 25581242

[B71] Silvera D. H. Lavack A. M. Kropp F. (2008). Impulse buying: the role of affect, social influence, and subjective wellbeing. J. Consum. Market. 25, 23–33. 10.1108/07363760810845381

[B72] Singh K. Jha S. D. (2008). Positive and negative affect, and grit as predictors of happiness and life satisfaction. J. Indian Acad. Appl. Psychol. 34, 40–45.

[B73] Teufel S. (1999). Argumentative zoning: information extraction from scientific articles (Ph.D. thesis), Centre for Cognitive Science, University of Edinburgh, Edinburgh, United Kingdom.

[B74] Thompson R. J. Mata J. Jaeggi S. M. Buschkuehl M. Jonides J. Gotlib I. H. (2012). The everyday emotional experience of adults with major depressive disorder: examining emotional instability, inertia, and reactivity. J. Abnorm. Psychol. 121:819. 10.1037/a0027978 22708886 PMC3624976

[B75] Tsugawa S. Kikuchi Y. Kishino F. Nakajima K. Itoh Y. Ohsaki H. (2015). Recognizing depression from twitter activity, in Proceedings of the 33rd Annual ACM Conference on Human Factors in Computing Systems (ACM), 3187–3196. 10.1145/2702123.2702280

[B76] Watson D. (2000). Mood and Temperament. New York, NY: Guilford Press.

[B77] Watson D. Clark L. A. (1997). Extraversion and its positive emotional core, in Handbook of Personality Psychology (Amsterdam: Elsevier), 767–793. 10.1016/B978-012134645-4/50030-5

[B78] Watson D. Clark L. A. Tellegen A. (1988). Development and validation of brief measures of positive and negative affect: the panas scales. J. Pers. Soc. Psychol. 54:1063. 10.1037/0022-3514.54.6.1063 3397865

[B79] Yarkoni T. (2010). Personality in 100,000 words: a large-scale analysis of personality and word use among bloggers. J. Res. Pers. 44, 363–373. 10.1016/j.jrp.2010.04.001 20563301 PMC2885844

[B80] Youyou W. Kosinski M. Stillwell D. (2015). Computer-based personality judgments are more accurate than those made by humans. Proc. Natl. Acad. Sci. U.S.A. 112, 1036–1040. 10.1073/pnas.1418680112 25583507 PMC4313801

